# Growth, structure, phase transition, thermal properties, and structural dynamics of organic–inorganic hybrid [NH_3_(CH_2_)_5_NH_3_]ZnCl_4_ crystal

**DOI:** 10.1038/s41598-022-21464-1

**Published:** 2022-10-07

**Authors:** Ae Ran Lim, Jiung Cho

**Affiliations:** 1 Graduate School of Carbon Convergence Engineering, Jeonju University, Jeonju, 55069 Korea; 2 Department of Science Education, Jeonju University, Jeonju, 55069 Korea; 3 Institute Korea Basic Institute, Seoul Western Center, Seoul, 03759 Korea

**Keywords:** Materials science, Physics

## Abstract

In this study, the physicochemical properties of [NH_3_(CH_2_)_5_NH_3_]ZnCl_4_ crystals were investigated using X-ray diffraction (XRD), Fourier transform infrared spectroscopy, differential scanning calorimetry (DSC), thermogravimetric analysis, and nuclear magnetic resonance (NMR). The crystals at 300 K had a monoclinic structure with C2/c space group and lattice constants are *a* = 21.4175 Å, *b* = 7.3574 Å, *c* = 19.1079 Å, *β* = 120.5190°, and Z = 8. Three endothermic peaks at 256, 390, and 481 K were observed in the DSC curve. From the single-crystal XRD patterns, powder XRD patterns, and optical microscopy results based on the temperature change, the phase transition and melting temperatures were determined to be 390 and 481 K, respectively. NMR studies indicated no change in ^1^H chemical shifts, but a change in the chemical shifts for C2, located between C1 and C3 of the cation at 340 K. Increase in molecular motion caused an increase in the spin–lattice relaxation time, T_1ρ_, at low spinning rates, under magic-angle spinning rate conditions. This crystal showed a minor change in the N−H···Cl hydrogen bond, related to the coordination geometry of the ZnCl_4_ anion.

## Introduction

The fabrication of hybrid compounds has recently been reported as a major challenge in the context of developing ferroelastic semiconductors^1^. On the other hand, the success of single-crystal ferroelectric performance makes hybrid compounds suitable candidates for flexible and wearable devices^2,3^. The diammonium series of hybrid materials, [NH_3_(CH_2_)_*n*_NH_3_]*BX*_4_ (*n* = 2, 3, 4,…; *B* = Mn, Co, Cu, Zn, Cd; and *X* = Cl^−^, Br^−^), with 0-, and two-dimensional (2D) structures, have been extensively investigated recently^4–12^. Recently, studies of [NH_3_(CH_2_)_*n*_NH_3_]*BX*_*2*_*X′*_2_ containing different halogen ions were conducted by Abdel-Aal et al.^13–15^ The physicochemical properties of organic–inorganic hybrids depend on their organic cations, inorganic anion coordination geometry of the metal ions, and halogen ions, which allow the properties of hybrid perovskites to be tailored^5–8,16–20^. The organic cation of the hybrid complex determines the structural flexibility and nonlinear optical properties, whereas the inorganic anion determines the thermal and mechanical properties^21,22^. The organic and inorganic layers form infinite 0- or 2D structures, connected by N−H···Cl hydrogen bonds^8,9,23,24^. The [NH_3_(CH_2_)_*n*_NH_3_] organic chains extend along the longest axis, and are located between the inorganic layers. Structural rearrangement, due to conformational changes of the chains, becomes important for long-chain alkylene–diammonium complexes [NH_3_(CH_2_)_*n*_NH_3_]*BX*_4_, with *n* >  > 4^25^. Among them, [NH_3_(CH_2_)_5_NH_3_]ZnCl_4_ (1,5-pentane-diammonium tetrachlorozincate), containing [NH_3_(CH_2_)_5_NH_3_] cations and layered ZnCl_4_ anions (with Zn atoms surrounded by four Cl atoms to form the ZnCl_4_ tetrahedron), is an interesting hybrid compound.

Previous publications report various studies on [NH_3_(CH_2_)_5_NH_3_]*B*Cl_4_ (*B* = Mn, Co, Cu, Zn, Cd) crystals. Filloleau et al.^26^ and Kanel et al.^27^ reported the magnetic and optical properties, and electron paramagnetic resonance, for *B* = Cu. The synthesis and characterisation of [NH_3_(CH_2_)_5_NH_3_]CdCl_4_ was first discussed by Kind et al.^25^ where structural phase transitions were studied using ^35^Cl and ^2^D nuclear magnetic resonance (NMR), birefringence, dilatation, and optical-domain investigations. Negrier et al.^28^ evaluated the crystal structures via X-ray diffraction (XRD), and Raman scattering experiments. Influence of the cation-length of [NH_3_(CH_2_)_*n*_NH_3_]CdCl_4_ (*n* = 2, 3, and 4) crystals on their thermal and structural dynamics have also been recently reported^22^. For *B* = Co and Mn, studies on crystal growth, and structures, have been reported^10,29^, and their magnetic and thermal properties have been briefly studied. However, there are no reports on [NH_3_(CH_2_)_5_NH_3_]ZnCl_4_ single-crystal growth.

Recently, the development of solar cells based on organic–inorganic hybrid materials has progressed rapidly. CH_3_NH_3_Pb*X*_3_ (*X* = Cl, Br, and I) thin-film photovoltaic devices have been used as solar cells. Despite progress in the application of CH_3_NH_3_Pb*X*_3_ as hybrid solar cells, these perovskites readily decompose in humid air, and are toxic owing to the presence of Pb, necessitating the development of alternative environment-friendly hybrid perovskite solar cells. The development of new types of 0- and 2D-hybrid materials, [NH_3_(CH_2_)_*n*_NH_3_]*BX*_4_, has increased the urgency of structure, optical properties, and dynamics investigations^1,8,30^.

This study is the first to investigate the crystal structure, phase-transition temperature (T_C_), and thermodynamic properties of [NH_3_(CH_2_)_5_NH_3_]ZnCl_4_ by single-crystal X-ray diffraction (XRD), powder XRD, differential scanning calorimetry (DSC), and thermogravimetric analysis (TGA) experiments. Nuclear magnetic resonance (NMR) chemical shifts, and spin–lattice relaxation times, T_1ρ_, for ^1^H, and ^13^C, were conducted using the magic-angle spinning (MAS) method, to analyse the coordinational geometry and molecular dynamics of the organic [NH_3_(CH_2_)_5_NH_3_] cation near T_C_. Static ^14^N NMR spectra were measured, with increase in temperature, to elucidate the atomic configurations of the cation. The change in coordinational geometry of this crystal with temperature was explained by the cation, and tetrahedral ZnCl_4_ anion. Thus, this work, correlating physicochemical and thermal properties, and structural dynamics, to the phase-transition mechanism, could facilitate practical applications of environment-friendly [NH_3_(CH_2_)_5_NH_3_]ZnCl_4_ crystals.

## Methods

To obtain [NH_3_(CH_2_)_5_NH_3_]ZnCl_4_ single crystals, an aqueous solution containing NH_2_(CH_2_)_5_NH_2_.2HCl (Aldrich, 98%), and ZnCl_2_ (Aldrich, 99.9%), was slowly evaporated in a thermostat, at 300 K. Single crystals were grown for approximately 3 weeks to a size 6 × 2 × 1.5 mm; they were colourless and transparent.

Fourier transform infrared (FT-IR) spectra, in the 4000–1000 cm^−1^ range, were measured using an FT-IR spectrometer (Perkin Elmer, L1600300) with a compressed KBr pellet.

The lattice parameters at various temperatures were determined by single-crystal XRD at the Seoul Western Center of the Korea Basic Science Institute (KBSI). A colorless crystal block was picked up with paratone oil and mounted on a Bruker D8 Venture PHOTON III M14 diffractometer equipped with a graphite-monochromated Mo-Kα (λ = 0.71073 Å) radiation source and a nitrogen cold stream (− 50 °C). Data was collected and integrated using SMART APEX3 (Bruker 2016) and SAINT (Bruker, 2016). The absorption was corrected by a multi-scan method implemented in SADABS. The structure was solved using direct methods and refined by full-matrix least-squares on *F*^2^ using SHELXTL. All non-hydrogen atoms were refined anisotropically, and the hydrogen atoms were added to their geometrically ideal positions. Additionally the powder XRD patterns of the [NH_3_(CH_2_)_6_NH_3_]ZnBr_4_ crystals were measured at several temperatures using an XRD system with a Mo-Kα radiation source. Experimental conditions were similar to a previously described method^22^.

DSC measurements were performed on a DSC instrument (25, TA Instruments), at heating and cooling rates of 10 °C/min, in the 200–570 K temperature range, under a flow of dry nitrogen gas. And, the optical observations were measured using an optical polarizing microscope within the temperature range of 300–573 K, with a Linkam THM-600 heating stage.

TGA was also performed at 10 °C/min heating rate, in the 300–873 K temperature range, under nitrogen gas.

The NMR spectra of the [NH_3_(CH_2_)_5_NH_3_]ZnCl_4_ crystals were measured using a Bruker 400 MHz Avance II + NMR spectrometer, at the same facility, KBSI. The Larmor frequency for ^1^H MAS NMR was ω_o_/2π = 400.13 MHz, and that for the ^13^C MAS NMR experiment was ω_o_/2π = 100.61 MHz. To minimise the spinning sideband, the MAS rate for ^1^H was measured at 10 kHz, whereas the MAS rate for ^13^C was measured at 5 kHz, and 10 kHz. Tetramethylsilane (TMS) was used as reference material for accurate NMR chemical-shift measurements. T_1ρ_ values were obtained using a π/2 − *τ* pulse, followed by a spin-lock pulse of duration *τ*, and the π/2 pulse-widths for ^1^H and ^13^C were measured by a previously published method^31^. Static ^14^ N NMR spectra were measured with a Larmor frequency of ω_o_/2π = 28.90 MHz, using the one-pulse method, with resonance frequency referenced using NH_4_NO_3_ as standard material.

## Experimental results

### FT-IR spectra

**Figure 1:**
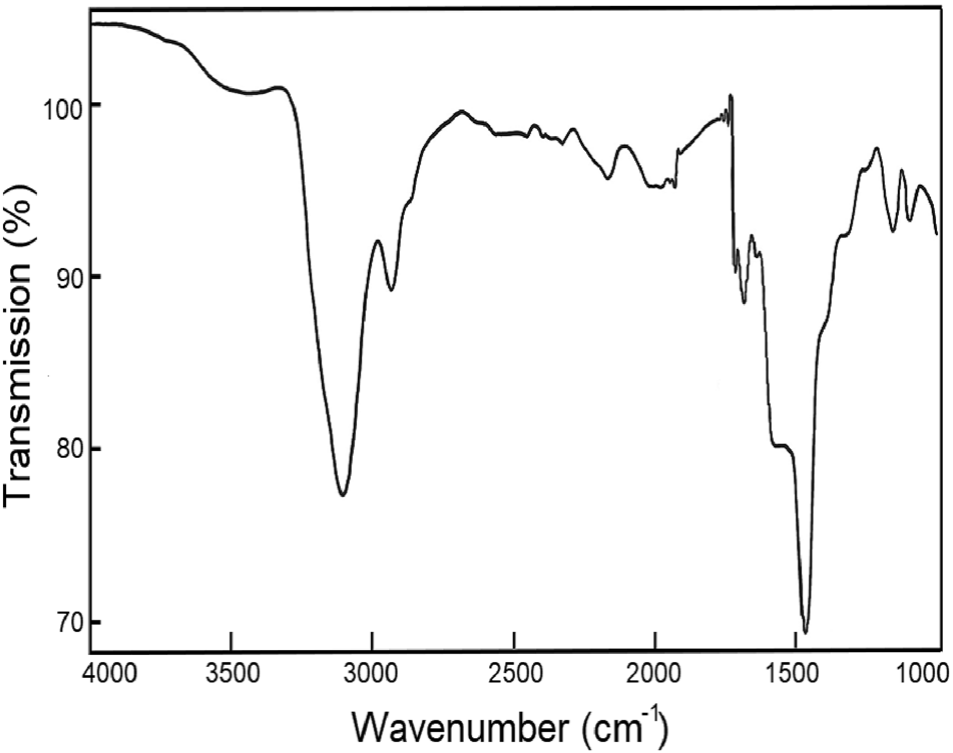
FT-IR spectrum of [NH_3_(CH_2_)_5_NH_3_]ZnCl_4_, in the 4000–1000 cm^−1^ range, at room temperature.

The FT-IR spectrum, in the 4000–1000 cm^−1^ range, was recorded at room temperature, and the results are shown in Fig. 1. The very strong peak near 3109 cm^−1^ is assigned to the C–H mode, and the peak at 2942 cm^−1^ is due to the N−H···Cl hydrogen bond. The spectral peaks at 1590 cm^−1^, and 1469 cm^−1^, correspond to the asymmetric deformation of NH_3_, and symmetric deformation of NH_3_, respectively. The peak near 1154 cm^−1^ is assigned to the C−N mode.

### Crystal structure

**Figure 2:**
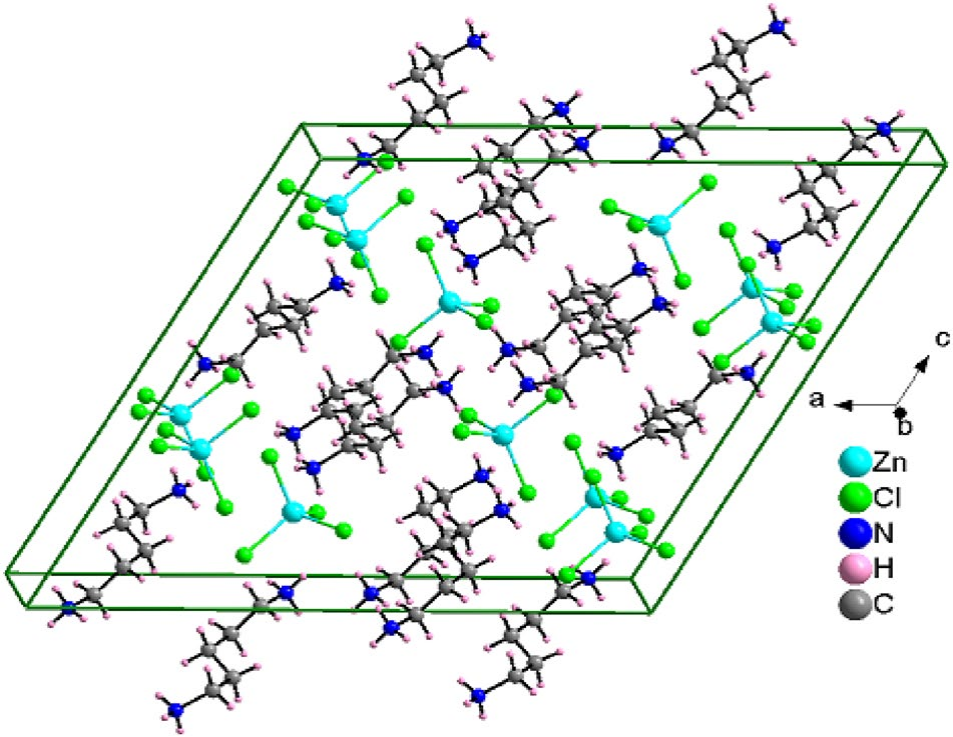
Crystal structure of [NH_3_(CH_2_)_5_NH_3_]ZnCl_4_ at 300 K.

Single-crystal XRD patterns for [NH_3_(CH_2_)_5_NH_3_]ZnCl_4_ crystals were obtained at 240, 300, and 350 K. The hybrid at 300 K was found to have crystallized as a monoclinic system with a C2/c space group and had lattice constants *a* = 21.4175 Å, *b* = 7.3574 Å, *c* = 19.1079 Å, *β* = 120.5190°, and Z = 8 (CCDC number: 2,170,368). Table 1 lists single-crystal XRD and refinement data of the [NH_3_(CH_2_)_5_NH_3_]ZnCl_4_ crystal, and Fig. 2 shows its structure. The Zn atom is coordinated by four Cl atoms, forming a nearly regular tetrahedron of ZnCl_4_.

**Table 1:** Crystal data and structure refinement for [NH_3_(CH_2_)_5_NH_3_]ZnCl_4_ at 300 K

Chemical formula	C_5_H_16_N_2_ZnCl_4_
Weight	311.37
Crystal System	Monoclinic
Space group	C2/c
T (K)	300
*a* (Å)	21.4175
*b* (Å)	7.3574
*c* (Å)	19.1079
β (°)	120.5190
Z	8
V (Å^3^)	2593.8
Radiation type	Mo-Kα
Wavelength	0.71073
Reflections collected	25,987
Independent reflections	3219 (*R*_int_ = 0.0268)
Goodness-of-fit on *F*^2^	1.058
Final *R* indices [I > 2sigma(I)]	*R*_1_ = 0.0211, *wR*_2_ = 0.0513
*R* indices (all data)	*R*_1_ = 0.0248, *wR*_2_ = 0.0531

The full data are available in the CIF files.

**Figure 3:**
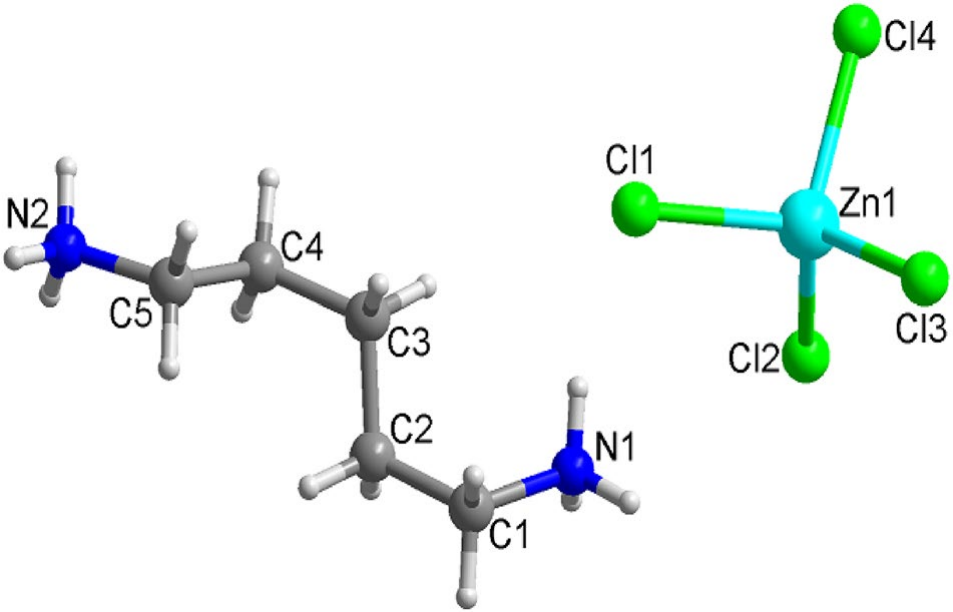
Thermal ellipsoid plot (50% probability) for structure of [NH_3_(CH_2_)_5_NH_3_]ZnCl_4_.

The hydrogen atoms of each formula unit are able to form hydrogen bonds N−H···Cl. The atomic numbering scheme and thermal ellipsoids for the H atoms are shown in Fig. 3, and their bond lengths and angles are summarized in Table 2. The lattice constants and structure were unchanged between 240 and 350 K. The crystallographic parameters at 240, 300, and 350 K are shown in the Supplementary Information.

**Table 2:** Bond-lengths (Å) and Bond-angles (°) at 300 K.

Bond-length (Å) and Bond-angle (°)			
Cl(1)–Zn(1)	2.2970 (4)	Cl(2)–Zn(1)–Cl(3)	114.228 (18)
Cl(2)–Zn(1)	2.2556 (4)	Cl(2)–Zn(1)–Cl(4)	110.246 (18)
Cl(3)–Zn(1)	2.2632 (4)	Cl(3)–Zn(1)–Cl(4)	106.651 (16)
Cl(4)–Zn(1)	2.2841 (4)	Cl(2)–Zn(1)–Cl(1)	108.102 (18)
		Cl(3)–Zn(1)–Cl(1)	110.719 (16)
		Cl(4)–Zn(1)–Cl(2)	106.641 (16)
N(1)–C(1)	1.490 (2)		
N(2)–C(5)	1.476 (2)		
C(1)–C(2)	1.506 (3)		
C(2)–C(3)	1.515 (3)		
C(3)–C(4)	1.527 (2)		
C(4)–C(5)	1.506 (2)		

**Figure 4:**
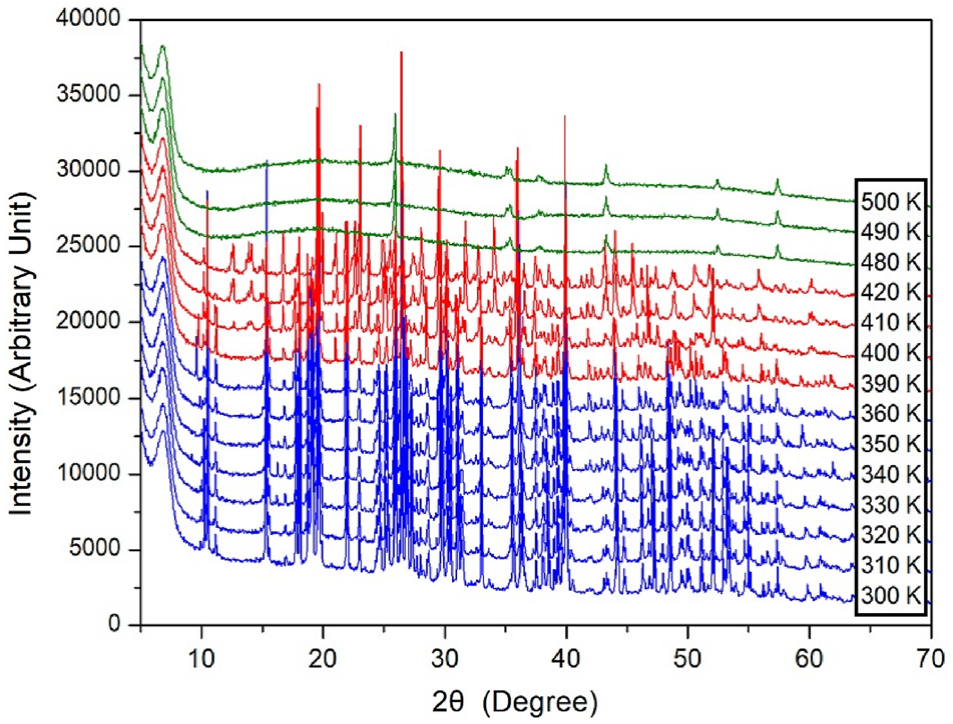
XRD powder patterns of [NH_3_(CH_2_)_5_NH_3_]ZnCl_4_ at several temperatures.

To confirm the phase transition temperature, the XRD powder-pattern experiments of the [NH_3_(CH_2_)_5_NH_3_]ZnCl_4_ crystal were carried out during heating, and the results, in the measuring range (2θ) of 7–65°, are shown in Fig. 4. The XRD patterns between 300 and 360 K, shown in blue, are nearly identical, and the peak at 9.6° in the powder XRD pattern recorded at 360 K was slightly different from that recorded at 300 K. The XRD patterns recorded above 390 K, shown in red, are more noticeable, with new peaks between 10 and 15° compared to those shown below 360 K, and this difference is related to the phase transition. The XRD pattern recorded above 480 K shows the peak from the alumina substrate. These peaks are in good agreement with the previously reported results for Al in a-Al_2_O_3_^32^; thus, it was confirmed that the single crystal melted and merged with the substrate.

### Phase transition

**Figure 5:**
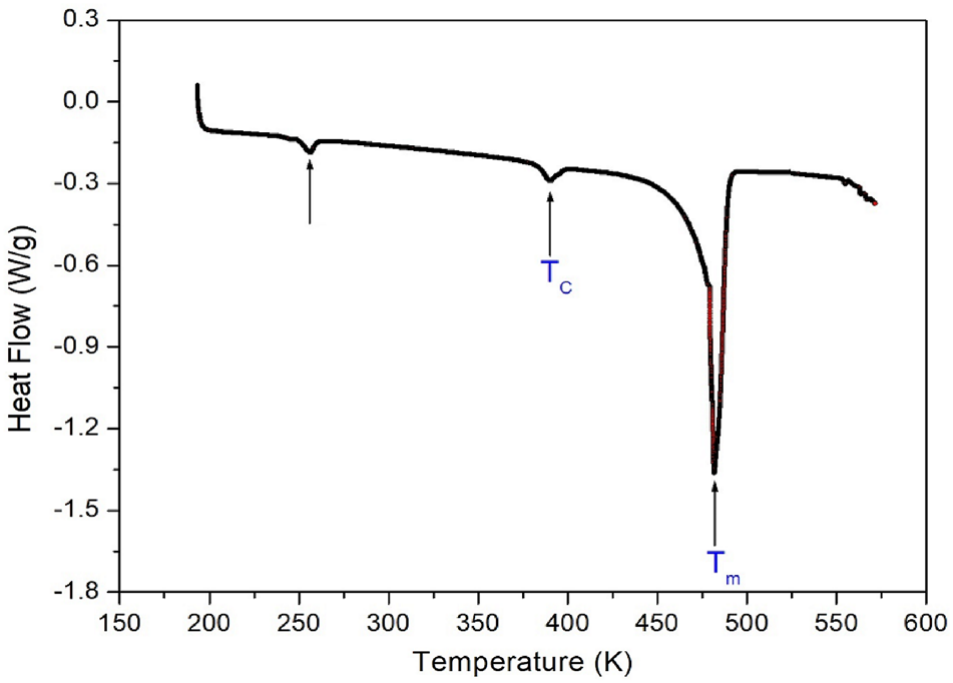
DSC curves for heating and cooling in [NH_3_(CH_2_)_5_NH_3_]ZnCl_4_.

The DSC experiment on the [NH_3_(CH_2_)_5_NH_3_]ZnCl_4_ crystal was carried out at a heating rate of 10 °C/min. After heating, as shown in Fig. 5, one of the three endothermic peaks at 481 K was very strong, whereas the other two peaks at 256 and 390 K, exhibited very weak intensities. The phase-transition temperatures shown in the DSC results were compared with those of the single-crystal XRD and powder XRD patterns. In addition, melting of a single crystal was observed using a polarizing microscope while increasing the temperature. When the temperature was increased from 300 to 450 K, the state of the single crystal did not change. However, it started to melt slightly when the temperature reached 460 K, and a significant portion of the single crystal melted at 500 K. Thus, the phase-transition temperature was determined as T_C_ = 390 K and the melting temperature was defined as T_m_ = 481 K. The lattice constants obtained from the single-crystal XRD results at 240 and 350 K were almost the same, and the structure showed a monoclinic. Thus, it was found that the small peak at 256 K in the DSC curve was independent of the phase transition.

### Thermal property

**Figure 6:**
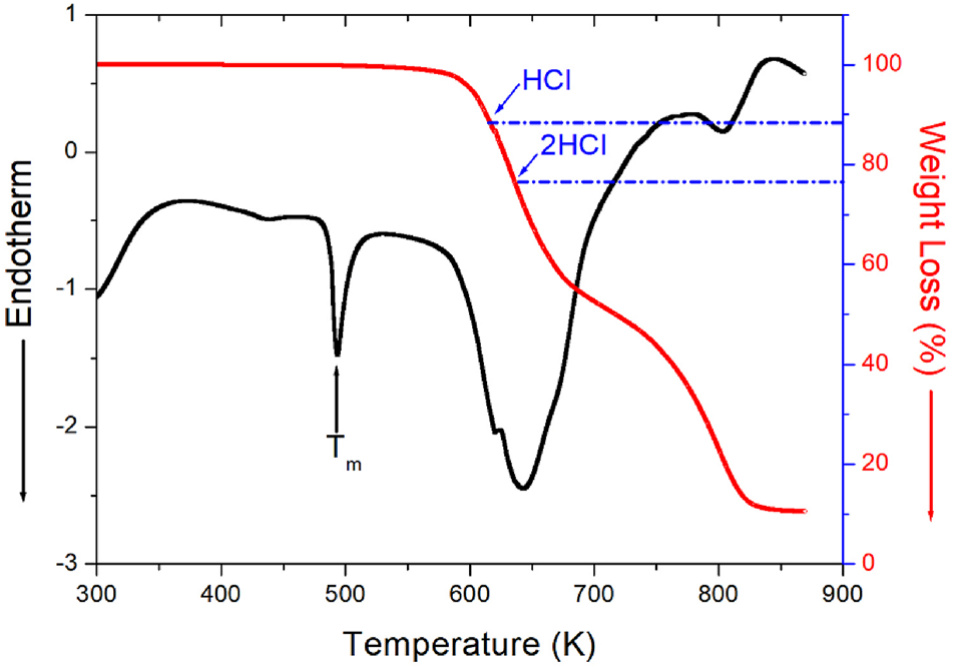
TGA and DTA curves of [NH_3_(CH_2_)_5_NH_3_]ZnCl_4_.

The TGA and DTA results, measured at a heating rate of 10 °C/min, are shown in Fig. 6. As the temperature increased, the molecular weight of the [NH_3_(CH_2_)_5_NH_3_]ZnCl_4_ crystals decreased. The molecular weight loss began at approximately 585 K, indicating partial thermal decomposition. From the total molecular weight of 311.39 mg, the amounts of residue produced by the decomposition of HCl and 2HCl were calculated using Eqs. (1) and (2):^24^

1 $$ \left\{ {\left[ {{\text{NH}}_{{2}} \left( {{\text{CH}}_{{2}} } \right)_{{5}} {\text{NH}}_{{2}} \cdot {\text{HCl}}} \right]{\text{ZnCl}}_{{4}} + {\text{ HCl }}\left( {\text{g}} \right)} \right\}/\left[ {{\text{NH}}_{{3}} \left( {{\text{CH}}_{{2}} } \right)_{{5}} {\text{NH}}_{{3}} } \right]{\text{ZnCl}}_{{4}} = { 88}.{29 }\% $$

2 $$ \left\{ {\left[ {{\text{NH}}_{{2}} \left( {{\text{CH}}_{{2}} } \right)_{{5}} {\text{NH}}_{{2}} } \right]{\text{ZnCl}}_{{4}} + {\text{ 2HCl }}\left( {\text{g}} \right)} \right\}/\left[ {{\text{NH}}_{{3}} \left( {{\text{CH}}_{{2}} } \right)_{{5}} {\text{NH}}_{{3}} } \right]{\text{ZnCl}}_{{4}} = { 76}.{58 }\% $$

The molecular-weight losses of 12% and 23% were due to the decomposition of HCl, and 2HCl, respectively. We also observed two step decomposition processes: the first resulted in a weight loss of 45% near 685 K, and the second resulted in 90% weight loss near 825 K. The weight loss of 45% was mainly attributed to organic decomposition, whereas it when the weight loss was 90% can be considered as a case where inorganic is almost decomposed and only Zn. One endothermic peak at 492 K in the DTA curve was assigned to the melting temperature obtained from the DSC results.

### ^1^H NMR chemical shifts and spin–lattice relaxation times

**Figure 7:**
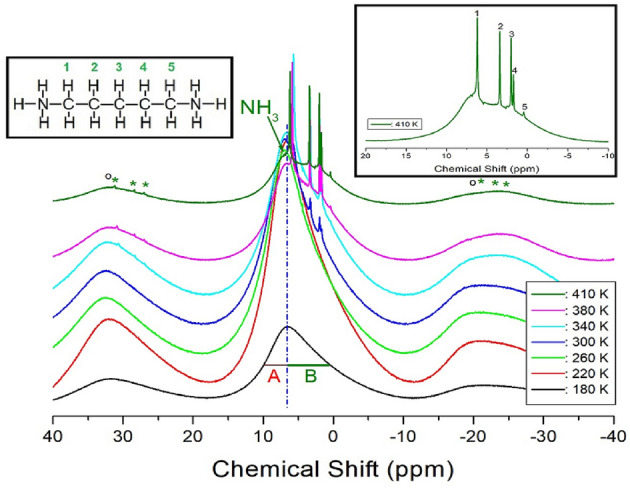
^1^H MAS NMR chemical shifts of [NH_3_(CH_2_)_5_NH_3_]ZnCl_4_ at several temperatures.

The ^1^H MAS NMR spectra of the [NH_3_(CH_2_)_5_NH_3_]ZnCl_4_ crystals were recorded as a function of temperature, and the ^1^H chemical shifts are shown in Fig. 7. Below 260 K, only one resonance line was observed, instead of two ^1^H signals from NH_3_ and CH_2_, and the NMR spectrum had an asymmetric shape. The linewidths, marked with A on the left, and B on the right, at the full-width-at-half-maximum (FWHM), were unequal. The asymmetric shape of the resonance line corresponded to the overlapping lines of ^1^H, in NH_3_ and CH_2_. At approximately 300 K, small and sharp signals started to appear in the right side due to the ^1^H signals of CH_2_. NH_3_ denotes ^1^H in NH_3_, and the remaining five signals represent ^1^H in 5 CH_2_, arbitrarily indicated by numbers 1, 2, 3, 4, and 5, as shown in the inset of Fig. 7. At 410 K, the ^1^H chemical shift for NH_3_ was recorded at 6.93 ppm, and those for 5 CH_2_ were obtained at 6.19, 3.40, 2.00, 1.72, and 0.44 ppm, respectively. Here, the spinning sidebands for the ^1^H signal in NH_3_ are marked with open circles, whereas those for the ^1^H signal in 5 CH_2_ are marked with asterisks. The ^1^H NMR chemical shifts for NH_3_ and CH_2_ were temperature-independent, indicating no change in the chemical environment around ^1^H with temperature.

**Figure 8:**
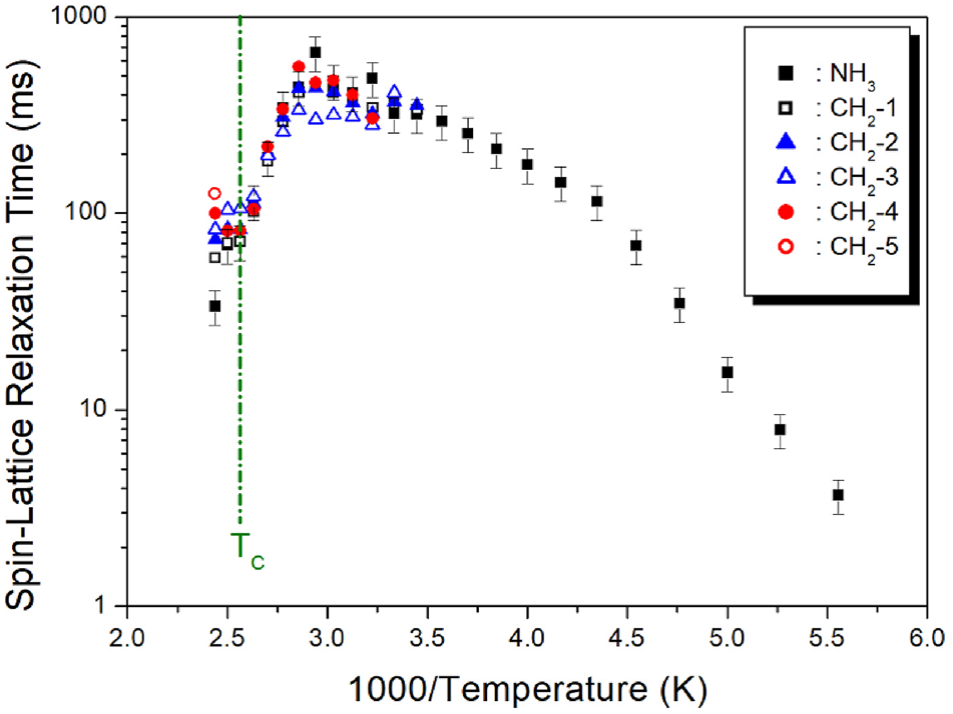
^1^H spin–lattice relaxation time of [NH_3_(CH_2_)_5_NH_3_]ZnCl_4_ as a function of inverse temperature.

The ^1^H MAS NMR spectra were measured by changing the delay time at each temperature, and the plots of spectral intensities, according to delay times, were expressed as mono-exponential curves. The recovery trace of the magnetisation is characterised by the spin–lattice relaxation time T_1ρ_, according to^33–35^

3 $$ I_{{\text{H}}} (\tau ) = I_{{\text{H}}} (0)\exp ( - \tau /T_{1\rho } ) $$

where I_H_ (*τ*) and I_H_ (0) are the signal intensities for the protons at time τ, and τ = 0, respectively. From Eq. (3), the ^1^H T_1ρ_ values were determined for NH_3_ and CH_2_, and the ^1^H T_1ρ_ results are shown in Fig. 8, as a function of the inverse temperature. The ^1^H T_1ρ_ values were strongly dependent on temperature change, in the order of 5–700 ms*.* As temperature increased, the ^1^H T_1ρ_ values of NH_3_ rapidly increased from 3.7 ms at 180 K, to 662 ms at 340 K. At 340 K, T_1ρ_ decreased again. No changes in the T_1ρ_ values were observed near T_C3_ and T_C2_. The ^1^H T_1ρ_ values of 5 CH_2_ in the [NH_3_(CH_2_)_5_NH_3_] cation were equal, within the error range. The ^1^H T_1ρ_ value at 340 K exhibited the highest value.

### ^13^C NMR chemical shifts and spin–lattice relaxation times

**Figure 9:**
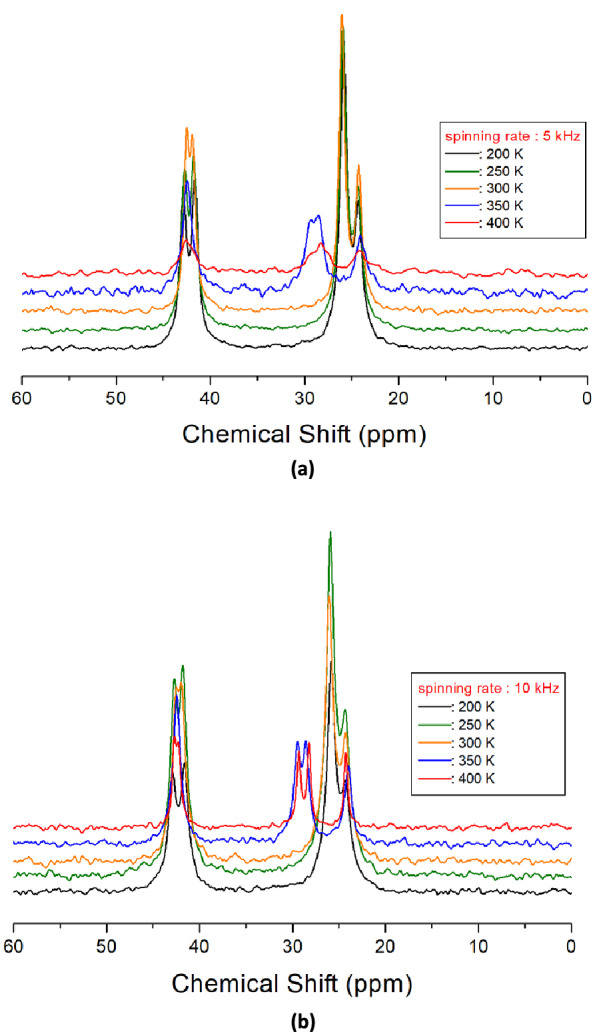
(**a**) ^13^C MAS NMR spectra of [NH_3_(CH_2_)_5_NH_3_]ZnCl_4_ at various temperatures, with a spinning rate of 5 kHz. (**b**) ^13^C MAS NMR spectra of [NH_3_(CH_2_)_5_NH_3_]ZnCl_4_ at various temperatures, with a spinning rate of 10 kHz.

**Figure 10:**
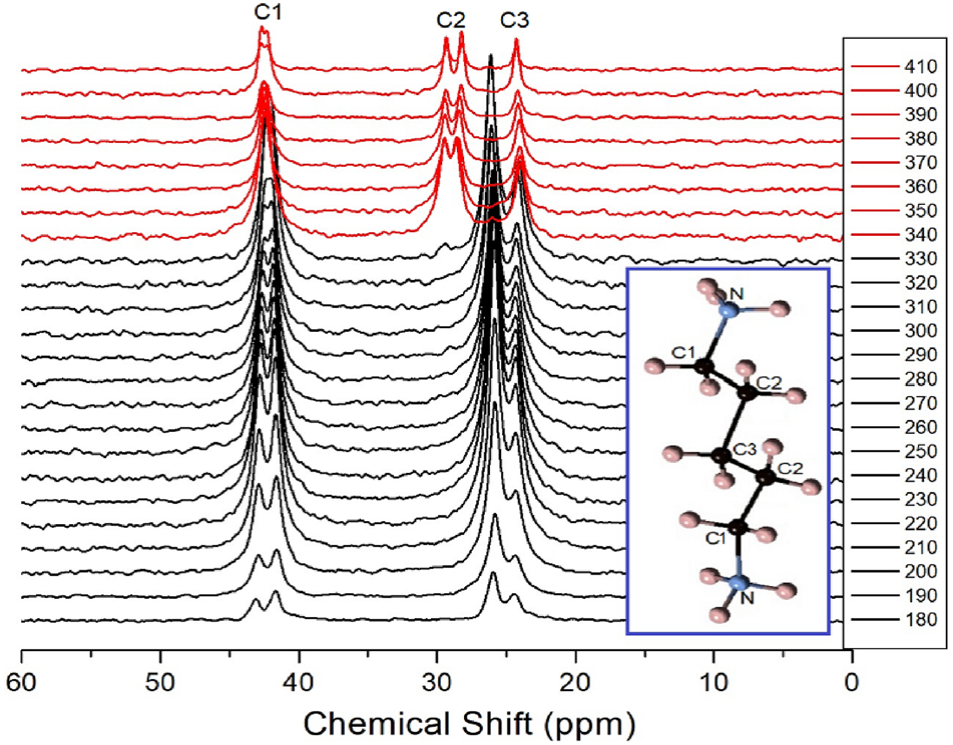
^13^C MAS NMR spectra of [NH_3_(CH_2_)_5_NH_3_]ZnCl_4_ above (red region), and below (black region) 340 K, with increasing temperature.

The ^13^C chemical shifts in the MAS NMR spectra for spinning rates of 5 kHz and 10 kHz are shown in Fig. 9a,b, repectively. The chemical shift for ^13^C in TMS at 300 K was recorded at 38.3 ppm, and this value was set as a standard reference value for ^13^C chemical shifts. For spinning rates of 5 kHz and 10 kHz, ^13^C chemical shifts at 200, 250, and 300 K were obtained, irrespective of the spinning rates, but the chemical shifts at 350 K and 400 K were not completely separated near 29 ppm, at 5 kHz spinning rate, as shown in Fig. 9a. The ^13^C chemical shifts with increasing temperature, at 10 kHz spinning rate, are shown in Fig. 10. In the [NH_3_(CH_2_)_5_NH_3_] cation structure, shown in Fig. 10, CH_2_ close to NH_3_ at both ends of the cation was labelled C1, CH_2_ at the centre of five CH_2_ chains was labelled C3, and CH_2_ between C1 and C3 was labelled C2. From 180 to 330 K, the chemical shifts showed slight change with temperature, but the chemical shifts at 340 K changed abruptly. At 300 K, ^13^C chemical shifts were recorded at 41.98 and 42.44 ppm for C1, 26.04 ppm for C2, and 24.32 ppm for C3, respectively, whereas those at 410 K were recorded at 42.68 and 42.83 ppm for C1, 28.19 and 29.29 ppm for C2, and 24.29 ppm for C3, respectively. C1 and C3 chemical shifts changed negligibly, but those of C2 in the cation changed completely. The spectrum recorded below 340 K is shown in black, and that recorded above 340 K is shown in red. The ^13^C chemical shifts did not change near T_C_ (= 390 K). The chemical shift of C2 changed rapidly at 340 K compared to those of C1 and C3. Thus, there is little structural change around C1 and C3 near 340 K, whereas the environment of C2 changes with temperature.

**Figure 11:**
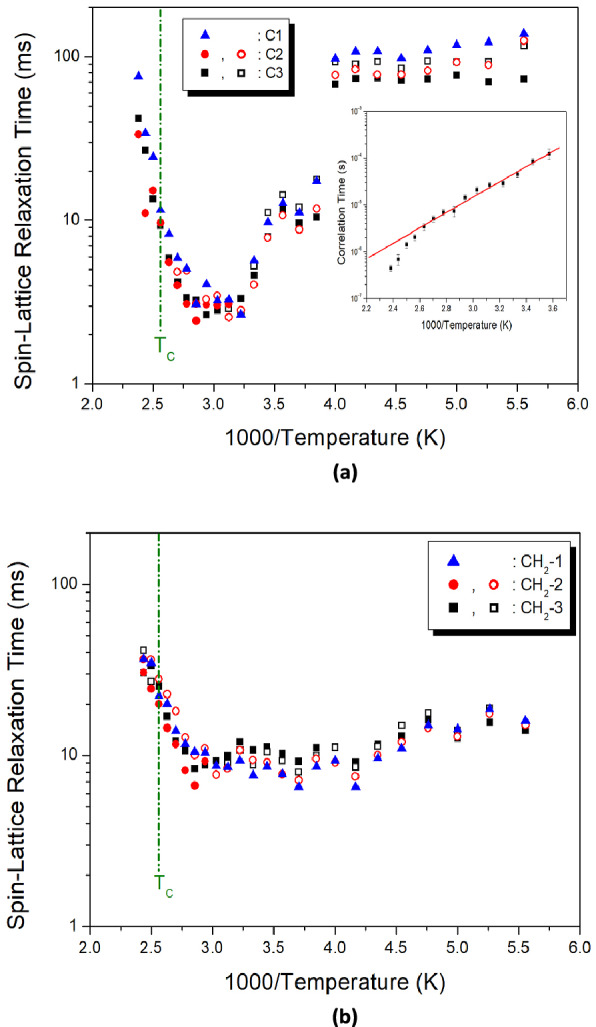
(**a**) ^13^C spin–lattice relaxation time of [NH_3_(CH_2_)_5_NH_3_]ZnCl_4_, as a function of inverse temperature, at 5 kHz spinning rate (Inset: Correlation times as a function of inverse temperature). (**b**) ^13^C spin–lattice relaxation time of [NH_3_(CH_2_)_5_NH_3_]ZnCl_4_, as a function of inverse temperature, at 10 kHz spinning rate.

The ^13^C MAS NMR spectra measured the change in intensity, with increasing delay time, at each temperature. The ^13^C T_1ρ_ values for C1, C2, and C3 were obtained at spinning rates of 5 kHz and 10 kHz. All the decay curves were described by a mono-exponential function, and the ^13^C T_1ρ_ values, from slopes of their recovery traces, were obtained as a function of 1000/T, as shown in Fig. 11a,b. At 5 kHz, ^13^C T_1ρ_ decreased slightly from 180 to 250 K, then decreased abruptly with increasing temperature, and increased again. Meanwhile, T_1ρ_, at 340 K, exhibited a minimum value of 2.64 ms, for C1. The minimum T_1ρ_ is due to the molecular motion of ^13^C in the [NH_3_(CH_2_)_5_NH_3_] cations. These T_1ρ_ values are described by the correlation time, τ_C_, for molecular motion; the T_1ρ_ value for molecular motion is given by^31,33^

4 $$ \begin{aligned} 1/T_{1\rho } & = \, (\gamma_{H} \gamma_{C} \hbar /r^{3} )^{2} \{ 4\tau_{C} /[1 \, + \omega_{1}^{2} \tau_{C}^{2} ] \, + \tau_{C} /[1 \, + \, (\omega_{H} - \omega_{C} )^{2} \tau_{C}^{2} ] \, + \, 3\tau_{C} \\ & \quad /[1 \, + \omega_{C}^{2} \tau_{C}^{2} ] \, + \, 6\tau_{C} /[1 \, + \, (\omega_{H} + \omega_{C} )^{2} \tau_{C}^{2} ] \, + \, 6\tau_{C} /[1 \, + \omega_{H}^{2} \tau_{C}^{2} ]\} \\ \end{aligned} $$

where γ_H_ and γ_C_ are the gyromagnetic ratios for ^1^H and ^13^C, respectively, *ħ* is Planck’s constant, *r* is the internuclear distance, ω_H_ and ω_C_ are the Larmor frequencies of ^1^H and ^13^C, respectively, and ω_1_ is the frequency of the spin-lock field (70.42 kHz). T_1ρ_ has a minimum value when ω_1_τ_C_ = 1. Therefore, the coefficient in Eq. (4) can be obtained from the relationship between T_1ρ_ and ω_1_. The correlation time, τ_C_, for molecular motion is calculated from the coefficient and T_1ρ_. The τ_C_, as a function of temperature, is expressed by the Arrhenius equation:^31,33^

5 $$ \tau_{{\text{C}}} = \tau_{{\text{o}}} {\text{exp}}( - {\text{E}}_{{\text{a}}} /{\text{k}}_{{\text{B}}} {\text{T}}) $$

where E_a_ is the activation energy of motion and k_B_ is the Boltzmann constant. E_a_ for C1 was obtained from the slope of log τ_C_ versus 1000/T, at temperatures above 250 K, as shown in the inset of Fig. 11a, and the value was 31.30 ± 1.23 kJ/mol. The E_a_ values for C2 and C3 were the same as those for C1, within the error range.

For 10 kHz spinning rate, the ^13^C T_1ρ_ values are shown in Fig. 11b, as a function of inverse temperature. The T_1ρ_ values initially decreased slightly as the temperature increased, and subsequently increased. No change was observed in the T_1ρ_ value near T_C_, but T_1ρ_ had a small minimum at 340 K, similar to the 5 kHz spinning-rate result. The T_1ρ_ values obtained at 5 kHz and 10 kHz spinning rates exhibited different trends. The T_1ρ_ values for ^13^C in the organic [NH_3_(CH_2_)_5_NH_3_] cation were strongly spinning-rate dependent. This is in agreement with a previous report by Gil and Alberti^36^, stating that T_1ρ_ values are spinning-rate dependent. The effects of molecular motion on T_1ρ_, and its spinning-rate dependence, are shown in Fig. 11a,b. At low temperatures, for 5 kHz and 10 kHz spinning rates, the T_1ρ_ values were significantly different, and at high temperatures, molecular motion was more active at low spinning-rates.

Therefore, T_1ρ_ dependence is very sensitive to intrinsic mobility, and a relative increase in T_1ρ_, at low spinning rates, decreases the correlation time, τ_C_.

### Static ^14^N resonance frequency

**Figure 12:**
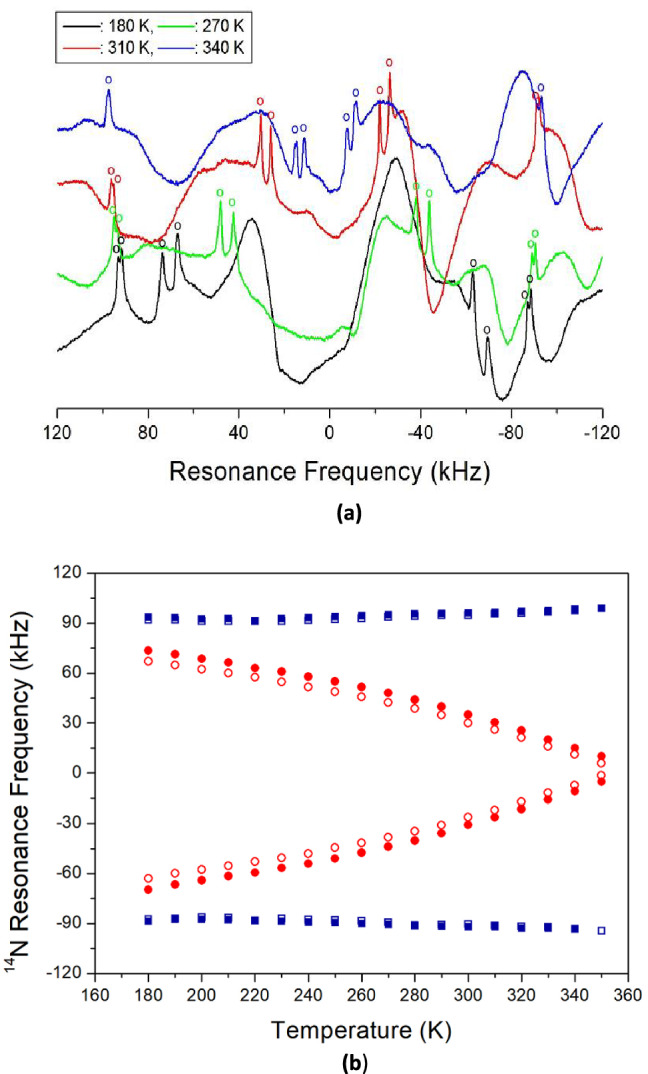
(**a**) In-situ ^14^N resonance frequency of [NH_3_(CH_2_)_5_NH_3_]ZnCl_4_ single crystal at various temperatures. (**b**) ^14^N resonance frequency of [NH_3_(CH_2_)_5_NH_3_]ZnCl_4_, as a function of temperature.

Static ^14^ N NMR experiments on [NH_3_(CH_2_)_5_NH_3_]ZnCl_4_ single crystals were carried out in the 180–430 K temperature range. As the spin number of ^14^N is I = 1, two resonance lines are expected from quadrupole interactions^32^. The ^14^N NMR spectrum is shown in Fig. 12a, at several temperatures. Recording ^14^N NMR signals at 28.90 MHz frequency was challenging owing to base-line wiggling. In the spectrum shown in Fig. 12a, the ^14^N signals are indicated by open circles. The detailed ^14^N NMR spectrum, according to temperature change, is shown in Fig. 12b. The eight lines of the four groups in the graph are attributed to the four inequivalent NH_3_. Four resonance lines of two pairs decreased with increasing temperature (red circle), whereas four resonance lines of the other two pairs slightly increased with increasing temperature (blue circle). The same pairs, for ^14^N, are indicated by symbols of the same colour. The ^14^N signals were difficult to observe at temperatures above 350 K. Continuous change in the ^14^N resonance frequency with temperature change indicated change in the coordination geometry of the environment around N, implying a change in the quadrupole coupling constant, e^2^qQ/h.

## Conclusion

The physicochemical properties of [NH_3_(CH_2_)_5_NH_3_]ZnCl_4_ crystals were investigated using single-crystal XRD, powder XRD, FT-IR, DSC, TGA, and NMR analyses. It was discovered that the crystals belong to a monoclinic system with a C2/c space group at 300 K, and the lattice constants are *a* = 21.4175 Å, *b* = 7.3574 Å, *c* = 19.1079 Å, *β* = 120.5190°, and Z = 8. The thermal decomposition temperature was relatively high (585 K), and the phase-transition temperature obtained from the DSC and XRD results was 390 K. It was found from single-crystal XRD and powder XRD results that 256 K, which was shown as a small DSC peak, was not a phase-transition temperature. In the NMR spectra, no change was observed in the ^1^H chemical shift, whereas a change in the chemical shift for C2, located between C1 and C3 of the cation, was observed at 340 K. This indicated change in the environment around C2 near 340 K. Additionally, changes in ^1^H and ^13^C T_1ρ_ values, indicating energy transfer, were observed near 340 K. Increase in molecular motion increased T_1ρ_, at low spinning rates, under MAS conditions. Additionally, the change of ^1^H T_1ρ_ values, ^13^C chemical shifts, and ^13^C T_1ρ_ values near 340 K was not related to the phase transition temperature.

In agreement with a previous report^22,31^ on metal ion *B* = Mn, Cu, or Cd,^8,9,23^ the ^1^H chemical shifts in NH_3_, and the influence of C1, located close to NH_3_, were large, indicating a large change in N−H···Cl. However, when *B* = Zn and Co^24^, the ^1^H chemical shifts in NH_3_ and CH_2_ exhibited little change, and the change in C1, located close to NH_3_, was almost identical to the changes of C2 and C3. Thus, [NH_3_(CH_2_)_5_NH_3_]ZnCl_4_ showed a minor change in the N−H···Cl hydrogen bond, associated with the inorganic ZnCl_4_ anion, whereas [NH_3_(CH_2_)_*n*_NH_3_]*B*Cl_4_ (*B* = Cu, Cd), as reported previously^22,31^, exhibited a significant change in the N−H···Cl hydrogen bond, related to the coordination geometry of the CuCl_4_ anion, with changing temperature.

The structural characterization, phase transition, thermodynamic properties, coordination geometry, and molecular motion of [NH_3_(CH_2_)_5_NH_3_]ZnCl_4_, explored in this study, could facilitate future research on environment-friendly organic–inorganic hybrid perovskites, for potential applications.

## Supplementary Information

Supplementary Information.

## Data Availability

The datasets generated and/or analysed during the current study are available in the CCDC 2,170,368. For ESI and crystallographic data in CIF or other electronic format see https://doi.org/.
