# Supplementary material for: Growth, structure, phase transition, thermal properties, and structural dynamics of organic–inorganic hybrid [NH3(CH2)5NH3]ZnCl4 crystal
Source: Sci Rep. 2022 Oct 7;12:16901. doi: 10.1038/s41598-022-21464-1 (PMC9546903; doi:10.1038/s41598-022-21464-1)
Supplement: Supplementary file 1 — Supplementary Information. [file 41598_2022_21464_MOESM1_ESM.rtf]

                    Supplementary Information


growth, structure, phase transition, thermal properties, and structural dynamics
of organic–inorganic hybrid [NH3(CH2)5NH3]ZnCl4 crystal: 


Ae Ran Lim1, 2, * & Jiung Cho3
1Graduate School of Carbon Convergence Engineering, Jeonju University, Jeonju 55069, Korea. 2Department of Science Education, Jeonju University, Jeonju 55069, Korea. email: arlim@jj.ac.kr  3Institute Korea Basic Institute, Seoul Western Center, Seoul 03759, Korea.


checkCIF/PLATON report 
Structure factors have been supplied for datablock(s) C5ZnCl4_240K_a
THIS REPORT IS FOR GUIDANCE ONLY. IF USED AS PART OF A REVIEW PROCEDURE FOR PUBLICATION, IT SHOULD NOT REPLACE THE EXPERTISE OF AN EXPERIENCED CRYSTALLOGRAPHIC REFEREE.
No syntax errors found.        CIF dictionary        Interpreting this report
Datablock: C5ZnCl4_240K_a 

Bond precision: 	C-C = 0.0030 A 	Wavelength=0.71073 	
Cell: 	a=21.294(2) b=7.3470(6) c=19.037(3) alpha=90 beta=120.390(3) gamma=90 	
Temperature: 	236 K 		
	Calculated 	Reported 	
Volume 	2569.1(5) 	2569.0(5) 	
Space group 	C 2/c 	C 2/c 	
Hall group 	-C 2yc 	-C 2yc 	
Moiety formula 	C5 H16 N2, Cl4 Zn 	C5 H16 N2, Cl4 Zn 	
Sum formula 	C5 H16 Cl4 N2 Zn 	C5 H16 Cl4 N2 Zn 	
Mr 	311.39 	311.37 	
Dx,g cm-3 	1.610 	1.610 	
Z 	8 	8 	
Mu (mm-1) 	2.702 	2.703 	
F000 	1264.0 	1264.0 	
F000' 	1271.13 		
h,k,lmax 	28,9,25 	28,9,25 	
Nref 	3195 	3190 	
Tmin,Tmax 	0.627,0.828 	0.626,0.746 	
Tmin' 	0.411 		
Correction method= # Reported T Limits: Tmin=0.626 Tmax=0.746
AbsCorr = MULTI-SCAN 
Data completeness= 0.998 	Theta(max)= 28.297 
wR2(reflections)=
R(reflections)= 0.0266( 2767) 
0.0653( 3190) 
S = 1.035 	Npar= 109 
The following ALERTS were generated. Each ALERT has the format        test-name_ALERT_alert-type_alert-level. Click on the hyperlinks for more details of the test.


PLAT911_ALERT_3_C Missing FCF Refl Between Thmin & STh/L=    0.600          2 Report


PLAT007_ALERT_5_G Number of Unrefined Donor-H Atoms ..............          6 Report
PLAT128_ALERT_4_G Alternate Setting for Input Space Group     C2/c       I2/a Note  
PLAT720_ALERT_4_G Number of Unusual/Non-Standard Labels ..........         11 Note  
PLAT794_ALERT_5_G Tentative Bond Valency for Zn1       (II)      .       1.96 Info  PLAT883_ALERT_1_G No Info/Value for _atom_sites_solution_primary .     Please Do !  
PLAT912_ALERT_4_G Missing # of FCF Reflections Above STh/L=  0.600          3 Note  
PLAT978_ALERT_2_G Number C-C Bonds with Positive Residual Density.          1 Info  

   0 ALERT level A = Most likely a serious problem - resolve or explain
0	ALERT level B = A potentially serious problem, consider carefully
1	ALERT level C = Check. Ensure it is not caused by an omission or oversight
   7 ALERT level G = General information/check it is not something unexpected
   1 ALERT type 1 CIF construction/syntax error, inconsistent or missing data
   1 ALERT type 2 Indicator that the structure model may be wrong or deficient
   1 ALERT type 3 Indicator that the structure quality may be low
   3 ALERT type 4 Improvement, methodology, query or suggestion
   2 ALERT type 5 Informative message, check

It is advisable to attempt to resolve as many as possible of the alerts in all categories. Often the minor alerts point to easily fixed oversights, errors and omissions in your CIF or refinement strategy, so attention to these fine details can be worthwhile. In order to resolve some of the more serious problems it may be necessary to carry out additional measurements or structure refinements. However, the purpose of your study may justify the reported deviations and the more serious of these should normally be commented upon in the discussion or experimental section of a paper or in the "special_details" fields of the CIF. checkCIF was carefully designed to identify outliers and unusual parameters, but every test has its limitations and alerts that are not important in a particular case may appear. Conversely, the absence of alerts does not guarantee there are no aspects of the results needing attention. It is up to the individual to critically assess their own results and, if necessary, seek expert advice.
Publication of your CIF in IUCr journals 
A basic structural check has been run on your CIF. These basic checks will be run on all CIFs submitted for publication in IUCr journals (Acta Crystallographica, Journal of Applied 
Crystallography, Journal of Synchrotron Radiation); however, if you intend to submit to Acta Crystallographica Section C or E or IUCrData, you should make sure that full publication checks are run on the final version of your CIF prior to submission.
Publication of your CIF in other journals 
Please refer to the Notes for Authors of the relevant journal for any special instructions relating to CIF submission.

PLATON version of 18/05/2022; check.def file version of 17/05/2022 
Datablock C5ZnCl4_240K_a - ellipsoid plot

Fig. S1. Thermal ellipsoid plot (50 % probability) for structure of [NH3(CH2)5NH3]ZnCl4 at 240 K.


checkCIF/PLATON report 
Structure factors have been supplied for datablock(s) ZnCl4_300K_a
THIS REPORT IS FOR GUIDANCE ONLY. IF USED AS PART OF A REVIEW PROCEDURE FOR PUBLICATION, IT SHOULD NOT REPLACE THE EXPERTISE OF AN EXPERIENCED CRYSTALLOGRAPHIC REFEREE.
No syntax errors found.        CIF dictionary        Interpreting this report
Datablock: ZnCl4_300K_a 

Bond precision: 	C-C = 0.0030 A 	Wavelength=0.71073 	
Cell: 	a=21.4175(12) b=7.3574(3) c=19.1079(16) alpha=90 beta=120.519(1) gamma=90 	
Temperature: 	300 K 		
	Calculated 	Reported 	
Volume 	2593.8(3) 	2593.8(3) 	
Space group 	C 2/c 	C 2/c 	
Hall group 	-C 2yc 	-C 2yc 	
Moiety formula 	C5 H16 N2, Cl4 Zn 	C5 H16 N2, Cl4 Zn 	
Sum formula 	C5 H16 Cl4 N2 Zn 	C5 H16 Cl4 N2 Zn 	
Mr 	311.39 	311.37 	
Dx,g cm-3 	1.595 	1.595 	
Z 	8 	8 	
Mu (mm-1) 	2.677 	2.677 	
F000 	1264.0 	1264.0 	
F000' 	1271.13 		
h,k,lmax 	28,9,25 	28,9,25 	
Nref 	3237 	3219 	
Tmin,Tmax 	0.549,0.725 	0.628,0.746 	
Tmin' 	0.388 		
Correction method= # Reported T Limits: Tmin=0.628 Tmax=0.746
AbsCorr = MULTI-SCAN 
Data completeness= 0.994 	Theta(max)= 28.320 
wR2(reflections)=
R(reflections)= 0.0211( 2910) 
0.0531( 3219) 
S = 1.058 	Npar= 110 
The following ALERTS were generated. Each ALERT has the format        test-name_ALERT_alert-type_alert-level. Click on the hyperlinks for more details of the test.


PLAT911_ALERT_3_C Missing FCF Refl Between Thmin & STh/L=    0.600         18 Report
PLAT913_ALERT_3_C Missing # of Very Strong Reflections in FCF ....          6 Note  


PLAT007_ALERT_5_G Number of Unrefined Donor-H Atoms ..............          6 Report
PLAT019_ALERT_1_G _diffrn_measured_fraction_theta_full/*_max < 1.0      0.998 Report
PLAT128_ALERT_4_G Alternate Setting for Input Space Group     C2/c       I2/a Note  
PLAT720_ALERT_4_G Number of Unusual/Non-Standard Labels ..........         11 Note  
PLAT794_ALERT_5_G Tentative Bond Valency for Zn1       (II)      .       1.96 Info  PLAT883_ALERT_1_G No Info/Value for _atom_sites_solution_primary .     Please Do !  
PLAT933_ALERT_2_G Number of HKL-OMIT Records in Embedded .res File          3 Note  
PLAT978_ALERT_2_G Number C-C Bonds with Positive Residual Density.          2 Info  

   0 ALERT level A = Most likely a serious problem - resolve or explain
   0 ALERT level B = A potentially serious problem, consider carefully
   2 ALERT level C = Check. Ensure it is not caused by an omission or oversight
   8 ALERT level G = General information/check it is not something unexpected
   2 ALERT type 1 CIF construction/syntax error, inconsistent or missing data
   2 ALERT type 2 Indicator that the structure model may be wrong or deficient
   2 ALERT type 3 Indicator that the structure quality may be low
   2 ALERT type 4 Improvement, methodology, query or suggestion
   2 ALERT type 5 Informative message, check

It is advisable to attempt to resolve as many as possible of the alerts in all categories. Often the minor alerts point to easily fixed oversights, errors and omissions in your CIF or refinement strategy, so attention to these fine details can be worthwhile. In order to resolve some of the more serious problems it may be necessary to carry out additional measurements or structure refinements. However, the purpose of your study may justify the reported deviations and the more serious of these should normally be commented upon in the discussion or experimental section of a paper or in the "special_details" fields of the CIF. checkCIF was carefully designed to identify outliers and unusual parameters, but every test has its limitations and alerts that are not important in a particular case may appear. Conversely, the absence of alerts does not guarantee there are no aspects of the results needing attention. It is up to the individual to critically assess their own results and, if necessary, seek expert advice.
Publication of your CIF in IUCr journals 
A basic structural check has been run on your CIF. These basic checks will be run on all CIFs submitted for publication in IUCr journals (Acta Crystallographica, Journal of Applied 
Crystallography, Journal of Synchrotron Radiation); however, if you intend to submit to Acta Crystallographica Section C or E or IUCrData, you should make sure that full publication checks are run on the final version of your CIF prior to submission.
Publication of your CIF in other journals 
Please refer to the Notes for Authors of the relevant journal for any special instructions relating to CIF submission.

PLATON version of 19/02/2022; check.def file version of 19/02/2022 
Datablock ZnCl4_300K_a - ellipsoid plot

Fig. S2. Thermal ellipsoid plot (50 % probability) for structure of [NH3(CH2)5NH3]ZnCl4 at 300 K.


checkCIF/PLATON report 
Structure factors have been supplied for datablock(s) C5ZnCl4_350K_a
THIS REPORT IS FOR GUIDANCE ONLY. IF USED AS PART OF A REVIEW PROCEDURE FOR PUBLICATION, IT SHOULD NOT REPLACE THE EXPERTISE OF AN EXPERIENCED CRYSTALLOGRAPHIC REFEREE.
No syntax errors found.        CIF dictionary        Interpreting this report
Datablock: C5ZnCl4_350K_a 

Bond precision: 	C-C = 0.0050 A 	Wavelength=0.71073 	
Cell: 	a=21.5350(8) b=7.3586(2) c=19.1648(11) alpha=90 beta=120.580(1) gamma=90 	
Temperature: 	350 K 		
	Calculated 	Reported 	
Volume 	2614.61(19) 	2614.61(19) 	
Space group 	C 2/c 	C 2/c 	
Hall group 	-C 2yc 	-C 2yc 	
Moiety formula 	C5 H16 N2, Cl4 Zn 	C5 H16 N2, Cl4 Zn 	
Sum formula 	C5 H16 Cl4 N2 Zn 	C5 H16 Cl4 N2 Zn 	
Mr 	311.39 	311.37 	
Dx,g cm-3 	1.582 	1.582 	
Z 	8 	8 	
Mu (mm-1) 	2.655 	2.655 	
F000 	1264.0 	1264.0 	
F000' 	1271.13 		
h,k,lmax 	28,9,25 	28,9,25 	
Nref 	3261 	3253 	
Tmin,Tmax 	0.632,0.830 	0.646,0.746 	
Tmin' 	0.418 		
Correction method= # Reported T Limits: Tmin=0.646 Tmax=0.746
AbsCorr = MULTI-SCAN 
Data completeness= 0.998 	Theta(max)= 28.319 
wR2(reflections)=
R(reflections)= 0.0308( 2591) 
0.0756( 3253) 
S = 1.030 	Npar= 109 
The following ALERTS were generated. Each ALERT has the format        test-name_ALERT_alert-type_alert-level. Click on the hyperlinks for more details of the test.


PLAT911_ALERT_3_C Missing FCF Refl Between Thmin & STh/L=    0.600          2 Report


PLAT007_ALERT_5_G Number of Unrefined Donor-H Atoms ..............          6 Report
PLAT128_ALERT_4_G Alternate Setting for Input Space Group     C2/c       I2/a Note  
PLAT720_ALERT_4_G Number of Unusual/Non-Standard Labels ..........         11 Note  
PLAT794_ALERT_5_G Tentative Bond Valency for Zn1       (II)      .       1.96 Info  PLAT883_ALERT_1_G No Info/Value for _atom_sites_solution_primary .     Please Do !  
PLAT912_ALERT_4_G Missing # of FCF Reflections Above STh/L=  0.600          7 Note  
PLAT978_ALERT_2_G Number C-C Bonds with Positive Residual Density.          0 Info  

   0 ALERT level A = Most likely a serious problem - resolve or explain
0	ALERT level B = A potentially serious problem, consider carefully
1	ALERT level C = Check. Ensure it is not caused by an omission or oversight
   7 ALERT level G = General information/check it is not something unexpected
   1 ALERT type 1 CIF construction/syntax error, inconsistent or missing data
   1 ALERT type 2 Indicator that the structure model may be wrong or deficient
   1 ALERT type 3 Indicator that the structure quality may be low
   3 ALERT type 4 Improvement, methodology, query or suggestion
   2 ALERT type 5 Informative message, check

It is advisable to attempt to resolve as many as possible of the alerts in all categories. Often the minor alerts point to easily fixed oversights, errors and omissions in your CIF or refinement strategy, so attention to these fine details can be worthwhile. In order to resolve some of the more serious problems it may be necessary to carry out additional measurements or structure refinements. However, the purpose of your study may justify the reported deviations and the more serious of these should normally be commented upon in the discussion or experimental section of a paper or in the "special_details" fields of the CIF. checkCIF was carefully designed to identify outliers and unusual parameters, but every test has its limitations and alerts that are not important in a particular case may appear. Conversely, the absence of alerts does not guarantee there are no aspects of the results needing attention. It is up to the individual to critically assess their own results and, if necessary, seek expert advice.
Publication of your CIF in IUCr journals 
A basic structural check has been run on your CIF. These basic checks will be run on all CIFs submitted for publication in IUCr journals (Acta Crystallographica, Journal of Applied 
Crystallography, Journal of Synchrotron Radiation); however, if you intend to submit to Acta Crystallographica Section C or E or IUCrData, you should make sure that full publication checks are run on the final version of your CIF prior to submission.
Publication of your CIF in other journals 
Please refer to the Notes for Authors of the relevant journal for any special instructions relating to CIF submission.

PLATON version of 18/05/2022; check.def file version of 17/05/2022 
Datablock C5ZnCl4_350K_a - ellipsoid plot

Fig. S3. Thermal ellipsoid plot (50 % probability) for structure of [NH3(CH2)5NH3]ZnCl4 at 350 K.
